# Osteoprotegerin expression in triple-negative breast cancer cells promotes metastasis

**DOI:** 10.1002/cam4.277

**Published:** 2014-06-28

**Authors:** Michael Weichhaus, Prabu Segaran, Ashleigh Renaud, Dirk Geerts, Linda Connelly

**Affiliations:** 1 Department of Pharmaceutical Sciences, College of Pharmacy, University of Hawaii at HiloHilo, Hawaii; 2 Department of Pediatric Oncology, Erasmus University Medical CenterRotterdam, The Netherlands

**Keywords:** Breast cancer, metastasis, osteoprotegerin, TRAIL

## Abstract

Osteoprotegerin (OPG) is a secreted member of the tumor necrosis factor (TNF) receptor superfamily that has been well characterized as a negative regulator of bone remodeling. OPG is also expressed in human breast cancer tissues and cell lines. In vitro studies suggest that OPG exerts tumor-promoting effects by binding to TNF-related apoptosis inducing ligand (TRAIL), thereby preventing induction of apoptosis. However, the in vivo effect of OPG expression by primary breast tumors has not been characterized. We knocked down OPG expression in MDA-MB-231 and MDA-MB-436 human breast cancer cells using shRNA and siRNA to investigate impact on metastasis in the chick embryo model. We observed a reduction in metastasis with OPG knockdown cells. We found that lowering OPG expression did not alter sensitivity to TRAIL-induced apoptosis; however, the OPG knockdown cells had a reduced level of invasion. In association with this we observed reduced expression of the proteases Cathepsin D and Matrix Metalloproteinase-2 upon OPG knockdown, indicating that OPG may promote metastasis via modulation of protease expression and invasion. We conclude that OPG has a metastasis-promoting effect in breast cancer cells.

## Introduction

At present, one in eight women in the United States will develop breast cancer 1. Recent advances in breast cancer detection and treatment have decreased the mortality rate of breast cancer 2, but rely largely on detection of the disease at early stages 3. A lack of knowledge regarding the molecular mechanisms underlying breast tumor progression to invasive and then metastatic disease limits our ability to treat advanced disease. The identification of factors that promote metastasis is essential for the development of new breast cancer therapies and a further reduction in breast cancer mortality.

In the current study, we have investigated the role of osteoprotegerin (OPG) in breast cancer metastasis. OPG, also called TNFRSF11B (NCBI GeneID: 4982), is a member of the tumor necrosis factor (TNF) receptor superfamily 4. It is a highly conserved glycoprotein which is secreted as a homodimer. OPG has mainly been characterized in its role as a negative regulator of osteoclast maturation, and thus an important mediator of bone remodeling 4,5. Bone homeostasis is maintained by the interplay between the receptor activator of nuclear factor kappa-B (NF-κB) called RANK, its activation ligand (RANKL) and OPG. Osteoclastogenesis is initiated by soluble RANKL binding to RANK expressed on the membrane of osteoclast precursors. OPG is also able to bind to RANKL, inhibiting activation of RANK and preventing osteoclast maturation 6.

OPG studies in the context of cancer have mainly focused on OPG acting in the bone microenvironment. The majority of advanced breast cancers invade bone, this can result in osteolytic metastases in which primary tumor cells are surrounded by osteoclasts degrading the bone tissue 7,8. As OPG is known to inhibit osteoclast maturation, recombinant OPG has been tested to treat breast carcinoma-related bone metastases by preventing osteolysis. A truncated form of OPG, Fc-OPG, showed inhibition of bone destruction in both murine and human studies 9,10. These studies investigate the impact of OPG that is either introduced or produced by bone cells and focus on actions within the bone microenvironment.

Recent studies have demonstrated OPG expression by primary human breast tumor cells and in breast cancer tissue samples 11–13. In a large cohort of invasive breast cancers (*n* = 400), 40% of samples showed OPG expression that was confined to tumor cells 11. In vitro studies show that OPG acts as a decoy receptor for TNF-related apoptosis inducing ligand (TRAIL) and can thereby block apoptosis 14,15. Indeed, OPG expression by breast cancer cells was sufficient to inhibit in vitro TRAIL-induced apoptosis 11,16. However, no in vivo studies have been performed that consider the role of endogenous OPG production by breast tumor cells out with the bone microenvironment.

To determine the in vivo significance of OPG production by the primary breast tumor*,* we knocked down OPG expression in MDA-MB-231 and MDA-MB-436 human breast cancer cells by shRNA or siRNA and measured the metastatic potential of these cells in vivo using the chick embryo metastasis model. We show that reduced OPG expression results in decreased metastasis of these human breast cancer cells. The OPG knockdown did not impact TRAIL sensitivity. However, OPG knockdown cells were less invasive and showed reduced expression of Cathepsin D and Matrix Metalloproteinase-2 (MMP-2), suggesting lower protease activity as a mechanism for the reduction in metastatic potential.

## Materials and Methods

### Cell culture and reagents

The human breast cancer cell lines MDA-MB-231 and MDA-MB-436 were purchased from the American Tissue Culture Collection (ATCC, Manassas, VA). Cells were maintained in Dulbecco's modification of Eagle's medium (DMEM), with 4.5 g/L glucose and sodium pyruvate without l-glutamine (Mediatech, Manassas, VA) supplemented with 10% (v/v) fetal bovine serum (FBS; Atlanta Biologicals, Lawrenceville, GA), 2 mmol/L l-Glutamine (Mediatech), and 50 *μ*g/mL Gentamicin (Life Technologies, Carlsbad, CA). Cells were kept at 37°C in humidified atmosphere containing 5% CO_2_.

### shRNA transfection

Human OPG-specific sequence 5′-CCTCCAAAGTACCTTCATTAT-3′ targeting nucleotides 396–416 of the human TNFRSF11B gene encoding OPG (defined by NCBI RefSeq NM_002546) or the nontargeting sequence 5′-CAACAAGATGAAGAGCACCAA-3′ (“SHC002,” negative control) were cloned into the pLKO.1 shRNA expression vector 17. pLKO.1 plasmids express 52 base pair shRNA molecules with 21-nucleotide mRNA specificity, driven by the efficient, ubiquitously active U6 snRNA promoter. For transfection, 2 × 10^6^ cells were plated in 100-mm tissue culture dishes with 10 mL DMEM and incubated overnight. For each plasmid, 14.5-*μ*g DNA was dissolved in 654 *μ*L OPTI-MEM (Life Technologies), after which 43.5-*μ*L FuGENE HD Transfection reagent (Promega, Madison, WI) was added according to the FuGENE HD protocol. Dishes were washed with 5 mL/dish phosphate-buffered saline (PBS) and supplemented with 15 mL/dish DMEM and the FuGENE/plasmid solution. After 24 h, cells were washed with PBS and medium was changed to DMEM containing 4-*μ*g/mL Puromycin selection antibiotic (Life Technologies). Transfected cells were continually maintained in selection medium.

### siRNA transfection

MDA-MB-231 or MDA-MB-436 cells (2 × 10^6^) were plated in 100-mm tissue culture dishes in 10-mL DMEM medium and incubated overnight. Two small interfering RNAs were prepared by aliquoting 2 × 500 *μ*L OPTI-MEM (Life Technologies) in 1.5-mL Eppendorf tubes for each siRNA. Ten-microliter lipofectamine (Life Technologies) or 10 *μ*L of 20 *μ*mol/L OPG siRNA (Stealth siRNAs for human OPG—HSS107349 [#1], HSS181651 [#2] and HSS181652 [#3])) or noncoding siRNA control (Stealth RNAi siRNA-negative control med GC; all from Life Technologies) were added to each OPTI-MEM aliquot and incubated for 2 min at room temperature. Lipofectamine and siRNA aliquots were combined and incubated for 20 min at room temperature. Meanwhile, dishes were washed once with 5 mL/dish PBS and once with 5 mL/dish OPTI-MEM. Dishes were then supplemented with 4 mL OPTI-MEM and 1 mL Lipofectamine/siRNA aliquot and incubated for 24 h at 37°C. After that incubation, dishes were washed once in PBS and supplemented with 10 mL/dish DMEM or used in further experiments.

### RNA isolation and quantitative Reverse Transcription PCR

After treatment, cells were washed with PBS and RNA extracted using the RNeasy MiniKit (Qiagen, Valencia, CA). Primary tumors were homogenized in a glass tissue grinder with homogenization buffer before extraction with the RNeasy MiniKit. RNA was quantified using a BioSpec-nano (Shimadzu Biotech, Columbia, MD). DNA contamination was removed by treating 8 *μ*g RNA with Ambion DNA-free DNase (Life Technologies), following the manufacturer's instructions. In a 20-*μ*L volume, 50 ng DNase-treated RNA was reverse transcribed using the iScript cDNA Synthesis Kit (Bio-Rad, Hercules, CA) on a C1000 Thermal Cycler (Bio-Rad), following the manufacturer's instructions with one cycle of 25°C for 5 min, 42°C for 30 min, and 85°C for 5 min. Next, 1 *μ*L of cDNA was amplified in a 25-*μ*L volume containing 1x iQ SYBR Green Supermix (Bio-Rad) with 400 nmol/L target-specific primer using a CFX96 quantitative PCR detection system (Bio-Rad) performing a hot-start PCR at 95°C for 3 min followed by 40 cycles of 95°C for 15 sec and 56°C for 40 sec. Relative fluorescent units (RFU) were plotted against cycle number using the Bio-Rad CFX Manager software (Bio-Rad). RFU is a quantitative measurement dependent on the amount of PCR product. The threshold cycle (C_t_) is the fractional cycle number at which the RFU exceeds a fixed level above baseline. These values for the target genes were normalized to GAPDH gene expression, obtaining ΔC_t_ = C_t_(GAPDH) – C_t_(target), relative differences were calculated as 2^ΔCt^. Each cDNA was amplified in triplicate for each target and control. RT negative controls included no template control and no reverse transcriptase control. Quantitative PCR negative control was no template control in triplicate for each target. Primer sequences are as follows: GAPDH sense: 5′-TCGACAGTCAGCCGCATCTTCTTT-3′, antisense: 5′-ACCAAATCCGTTTCCGACCTT-3′; OPG sense 5′-AACGGCAACACAGCTCACAAGAAC-3′ antisense: 5′-TGCTCGAAGGTGAGGTTAGCATGT-3′; MMP-2 sense: 5′-AGAAGGATGGCAAGTACGGCTTCT-3′, antisense: 5′-AGTGGTGCAGCTGTCATAGGATGT-3′, MMP-9 sense: 5′-TGACGGCTCACACTTGTAATCCCA-3′, antisense: 5′-TCAGCCTTCTGCATAG CTGGAACT-3′, and Cathepsin D sense: 5′-TTGCTG TTTTGTTCTGTGGTTTTC-3′, antisense 5′-CAGACAGGCAGGCAGC ATT-3′.

### Enzyme-linked immunosorbent assay

After treatments, cell culture media (supernatant) aliquots were collected for analysis. OPG concentrations were assessed using the Human OPG/TNFRSF11B DuoSet ELISA kit (R&D systems, Minneapolis, MN) according to the manufacturer's instructions. Each standard treatment was analyzed in triplicate.

### MTT (3-(4,5-dimethylthiazol-2-yl)-2,5-diphenyltetrazoliumbromide) assay

Five thousand cells/well were plated in 96-well plates in 100 *μ*L/well medium. After 24 h, medium was removed and wells were resupplemented with 100 *μ*L/well medium with or without TRAIL (5–500 ng/mL). Six replicates were performed for each condition. After 44 h, wells were supplemented with 1 mg/mL MTT (Biosynth AG, Staad, Switzerland). After 48-h incubation, medium was removed; wells were supplemented with 200 *μ*L/well DMSO and incubated in the dark for 15 min with gentle agitation. Absorbance was measured at 595 nm using a Bio-Rad iMark microplate reader.

### Invasion assay

MDA-MB-231 and MDA-MB-436 cells were transfected with OPG or control siRNA as described above. Cell invasion was measured using the Cultrex 96 Well Collagen IV Cell Invasion Assay (Trevigen, Gaithersburg, MD) 24 h after transfection. Each condition was analyzed in sestuplicates with three independent experiments.

### Chick embryo spontaneous metastasis assay

Chick embryo metastasis assays were performed according to the method described by Zijlstra and colleagues 18. Fertilized white leghorn chicken eggs (Goode Enterprises, Papaikou, HI) were incubated at 37°C with 70% humidity in a rotary incubator (1502 Sportsman; GQ Manufacturing, Savannah, GA) for 10 days. After this, the chorioallantoic membrane (CAM) underneath the eggshell was dropped by drilling a small hole in the air sac and a second close to the allantoic vein, not penetrating the membranes. A small incision was made into the eggshell membrane, leaving the CAM intact. A weak vacuum was used to drop the CAM and a 1 cm^2^ opening was cut in proximity to the second incision near the allantoic vein. The CAM was abraded with a sterile cotton swab to access the mesenchyme. Cells were suspended in 50% DMEM/50% matrigel (Basement Membrane Matrix, Growth Factor Reduced, BD Biosciences, San Jose, CA). A 25-*μ*L inoculum (containing 3 × 10^6^ cells) was pipetted onto the CAM and the hole was sealed with tape. The eggs were returned to a stationary incubator (1550 Hatcher; GQ Manufacturing) for an additional 7 days. After this incubation, the extra-embryonic tumor was excised and weighed. A sample of the lower CAM and the embryonic liver were harvested and analyzed for the presence of tumor cells by Alu PCR as described below.

### Chick embryo experimental metastasis assay

Fertilized white leghorn chicken eggs were incubated as described above but for 12 days. The allantoic vein was exposed by removing the overlaying eggshell without penetrating the eggshell membrane. The eggshell membrane was rendered transparent by applying a drop of mineral oil. A 1 cc insulin syringe with 28G½ needle (Becton Dickinson USA, Franklin Lakes, NJ) was used to inject 1 × 10^6^ cells in 100 *μ*L volume of PBS into the allantoic vein. The injection site was sealed with tape; the eggs were returned to a stationary incubator and incubated for an additional 7 days. After this incubation, chick embryo liver and lungs and a sample of the CAM distant from the injection site were harvested and analyzed for the presence of tumor cells by Alu PCR as described below.

### DNA extraction from chick tissue and quantitative PCR analysis of Alu repeat sequences

Genomic DNA from harvested tissue samples was extracted using the Puregene DNA purification system (Qiagen) according to the manufacturer's instructions. DNA was quantified using a BioSpec-nano (Shimadzu Biotech). Genomic Alu repeat sequences were amplified by quantitative PCR as described above in a 25-*μ*L volume containing 30 ng DNA, 1x iQ SYBR Green Supermix (Bio-Rad) and 400 nmol/L of human Alu primers (5′-ACGCCTGTAATCCCAGCACTT-3′ and 5′-TCGCCCAGGCTGGAGTGCA-3′). Amplification was carried out on the Bio-Rad CFX96 system by performing a hot-start PCR at 95°C for 2 min followed by 30 cycles of 95°C for 30 sec, 63°C for 30 sec, and 72°C for 30 sec. Genomic chick DNA was measured by amplifying chicken GAPDH in the same samples using specific primers (5′-GAGGAAAGGTCGCCTGGTGGATCG-3′ and 5′-GGTGAGGA CAAGCAGTGAGGAGCG-3′) using the same PCR condi tions as described for Alu. Quantification of PCR products was carried out as described above.

### Quantitative Reverse Transcription PCR array

MDA-MB-231 breast cancer cells transfected with OPG shRNA or control shRNA were plated at 2 × 10^6^ cells in 100-mm dishes in 10-mL medium for 24 h and RNA prepared as above. 1 *μ*g RNA was reverse transcribed using the RT^2^ First Strand Kit (Qiagen). The resulting cDNA was mixed with the RT^2^ SYBR Green Mastermix (Qiagen) and plated on the Human Breast Cancer RT² Profiler PCR Array (Qiagen). The array was analyzed on the Bio-Rad CFX96 system using PCR at 95°C for 10 min, followed by 40 cycles of 95°C for 15 sec, and 60°C for 60 sec. Results were analyzed using the online RT^2^ Profiler PCR Array Data Analysis Tool (Qiagen) to obtain fold-change and significance values.

### Public dataset analysis

The Breast Invasive Carcinoma (TCGA-2013) data set (934 samples) was queried at cBioportal (http://www.cbioportal.org) for OPG (TNFRSF11B) DNA copy number alterations and correlated patient survival. The TCGA-2013 and other breast cancer sets at Oncomine (http://www.oncomine.org) were queried for OPG DNA copy number alterations and mRNA expression values. All cBioportal and Oncomine analyses were performed at the standard settings provided. Clinical data and other cohort details are available at the websites. For the OPG mRNA expression differences between basal and luminal samples, and for the OPG-MMP2 expression correlation analyses, CEL data from the public Affymetrix U133A or U133 Plus 2.0 array (Santa Clara, CA) data sets for the Barretina-917 (GSE36133), Bild-19 (GSE3156), Chin-124 (E-TABM-158), Desmedt-55 (GSE16391), EXPO-351 (GSE2109), Garnett-727 (E-MTAB-783), Hoeflich-51 (GSE12777), Huang-46 (GSE6569), Loi-77 (GSE9195), and Zhang-136 (GSE12093) breast cancer sets were downloaded from http://www.ncbi.nlm.nih.gov/geo/for the GSE sets 19, and from http://www.ebi.ac.uk/arrayexpress/for the Chin-124 and Garnett-727 sets, and analyzed as described 20 using R2; an Affymetrix analysis and visualization platform developed in the Department of Human Genetics at the Academic Medical Center—University of Amsterdam (http://r2.amc.nl). Gene transcript levels were determined from data image files using GeneChip operating software (MAS5.0 and GCOS1.0; Affymetrix). Samples were scaled by setting the average intensity of the middle 96% of all probe set signals to a fixed value of 100 for every sample in the data set, allowing comparisons between microarrays. Only expression values with a significant p value (“present call”) were used. The R2 TranscriptView genomic analysis and visualization tool was used to check whether the probe set selected uniquely mapped to an antisense position in an exon of the gene. The probe sets selected for TNFRSF11B (204933_at) and for MMP2 (201069_at) met all these criteria showed the highest expression in samples containing a present call for that gene and were used for analysis. The secondary TNFRSF11B probe set (204932_at) showed similar results in all cases.

### Statistical analysis

**Figure 1:**
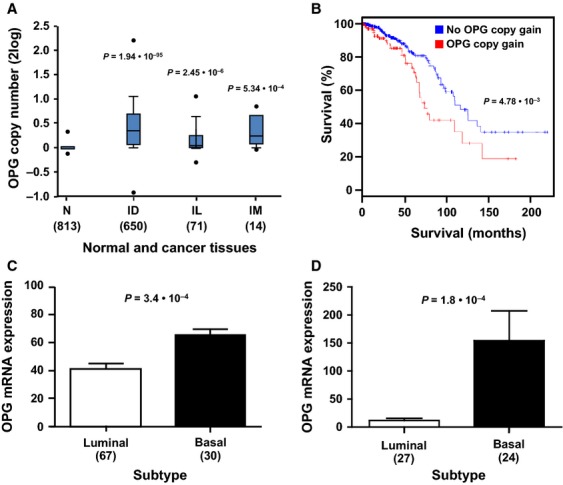
OPG DNA copy number and mRNA expression variations in human breast cancer. (A) OPG gene copy numbers in the TCGA-2013 breast cancer set (934 samples) were analyzed at the Oncomine website. The major invasive tumor subtypes display significantly higher OPG DNA copy numbers than normal breast tissue. N, normal breast tissue, ID, invasive ductal carcinoma, IL, invasive lobular carcinoma, IM, invasive mixed carcinoma (ductal and lobular). The *P* values are calculated with a Student's *t*-test. (B) The TCGA-2013 breast cancer set was analyzed at the cBioPortal website for the correlation of tumor OPG DNA copy number with patient outcome. A Kaplan–Meier analysis shows a significantly worse prognosis for patients with tumor OPG gene copy gain (red, 182 samples), compared to patients with tumor normal OPG gene copy numbers (blue, 752 samples; *P* = 4.8 × 10^−3^). The *P* value was calculated with a log-rank test. (C) OPG mRNA expression was compared between basal and luminal breast cancer subtypes at the R2 website. The Chin-124 breast cancer set, the largest data set annotated for these subtypes, shows significantly higher OPG levels in basal than in luminal samples (*P* = 3.4 × 10^−4^). (D) OPG mRNA expression was compared between basal and luminal breast cancer cell lines at the R2 website. Hoeflich-51, the largest breast cancer cell line data set (51 samples) with this annotation, shows significantly higher OPG levels in basal than in luminal cell lines (*P* = 1.8 × 10^−4^). The Barretina-917 cancer cell line set (with 49 breast cancer samples) showed a similar significant difference. In addition, the Garnett-727, Huang-46, and Bild-19 cancer cell lines sets showed the same trend, but without significant *P* values, probably because of the smaller amount of breast cancer samples in these data sets (38, 21, and 19, respectively). Higher OPG mRNA expression in luminal samples compared to basal samples was never found in any data set (results not shown). The *P* value in (C and D) was calculated with a Kruskal–Wallis test. In (A–D), the number of samples is in parentheses.

Statistical analyses were performed using Graph Pad Prism (GraphPad Software Inc., La Jolla, CA). All data are plotted graphically with vertical bars representing standard error. The unpaired Student's *t*-test was used to assess differences between OPG gene copy numbers in breast cancer and normal breast tissue samples (Fig.1A), and between experimental conditions. A log-rank test was used to determine significance of OPG gene amplification on overall survival (Fig.1B). The difference in OPG mRNA expression between basal and luminal samples was determined using the Kruskal–Wallis test (Fig.1C and D). Pearson (2-log) correlation was used to assess significance the OPG-MMP2 expression correlation values (Fig.6). A probability (*P*) value of less than 0.05 was taken as an appropriate level of significance.

## Results

### OPG DNA copy number gain and high mRNA expression are linked to aggressive breast cancer subtypes and poor outcome

To investigate a potential link between OPG expression and human breast cancer progression, we analyzed the largest breast cancer cohort in the public domain, the TCGA-2013 breast invasive carcinoma data set, through the cBioPortal website 21,22 (http://www.cbioportal.org). We found that OPG gene copy gain occurred in 182 of 934 tumors in the set (19.4%), but OPG gene copy loss only in 1 of 934 tumors (not shown). OPG gains, as the major OPG DNA copy number variation, suggest a tumor-supporting, “oncogenic,” role for OPG in breast cancer. Indeed, analysis of this breast cancer cohort at the Oncomine website (http://www.oncomine.org) revealed that OPG copy number gains occurred in the major invasive breast carcinoma subtypes present in the set (ductal, lobular, and mixed), and that OPG copy numbers were significantly higher than in normal breast tissue (Fig.1A). The presence of OPG copy number gain is consequently a significant predictor of decreased overall survival in this cohort (*P* = 4.78 × 10^−3^; Fig.1B).

OPG mRNA expression was analyzed in multiple breast cancer cohorts available at Oncomine and R2 (r2.amc.nl). Several of these tumor sets showed significantly higher OPG expression in tumor samples than in normal breast tissue, especially in invasive tumor subtypes, while in contrast tumors with lower OPG expression than in normal tissue were never apparent (not shown). These results again suggest a tumor-supporting role for OPG in breast cancer. Interestingly, OPG mRNA expression was higher in basal than in luminal tumor samples in breast cancer sets that were annotated for these tumor subtypes at R2. In the largest set, Chin-124, this difference is clearly significant (*P* = 3.4 × 10^−3^; Fig.1C). We also analyzed the largest breast cancer cell line set that describes basal and luminal characteristics, the Hoeflich-51 set. Here, OPG mRNA expression was significantly higher in basal than in luminal subtypes (*P* = 1.8 × 10^−4^; Fig.1D). This final analysis allowed us to select cell line models for the functional studies described below.

### OPG expression knockdown in MDA-MB-231 breast cancer cells reduces metastasis in the chick embryo spontaneous metastasis model

**Figure 2:**
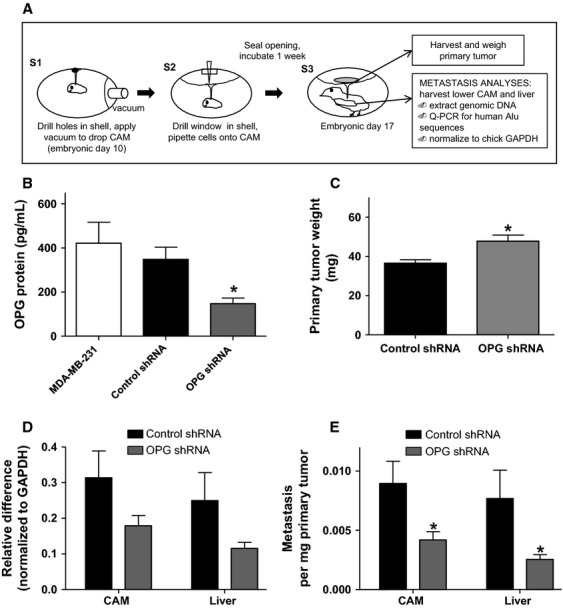
OPG knockdown by shRNA reduces metastasis of MDA-MB-231 cells in the chick embryo spontaneous metastasis model. (A) Overview of this model. (B) MDA-MB-231 cells were stably transfected with OPG shRNA or control shRNA. OPG expression was measured by ELISA in cell culture supernatants after 24-h incubation (*P < 0.05 significantly different from shRNA control cells, *n* ≥ 3). OPG shRNA or control shRNA cells were incubated 1 week in the chick embryo metastasis model and (C) primary tumors were collected and weighed (*P < 0.05 significantly different from shRNA control cells, *n* = 32) and (D) chick lower CAM and liver were analyzed by quantitative PCR for human Alu DNA (CAM; *P* = 0.0963 different from shRNA control cells, *n* = 46, liver; *P* = 0.0836 different from shRNA control cells, *n* = 42). (E) Quantitative PCR values for lower CAM and liver that were normalized to corresponding primary tumor weight (*P < 0.05, *n* = 42). All data are represented as mean ± SEM.

We used the chick embryo spontaneous metastasis model, outlined in Figure2A, to investigate the functional significance of OPG expression in vivo. In this model, a hole is drilled in the egg at embryonic day 10 (S1) and tumor cells are inoculated onto the CAM surrounding the developing chick embryo (S2). Upon incubation, a primary tumor forms on the CAM, allowing access to the chick vasculature for metastasis to more distant chick tissues (S3). Metastasis can be quantified by performing quantitative PCR for human Alu repeat sequences in DNA extracted from chick tissue 18. The chick embryo metastasis model offers considerable advantages over other in vivo systems. The developing chick provides a naturally immunodeficient host that will tolerate injection of human tumor cells. In addition, all the steps of the metastatic cascade are recapitulated: invasion and intravasation into the chick vasculature, travel in the circulation, and finally adherence and extravasation to form metastatic foci throughout the chick. Since our in silico analysis of breast cancer cells showed higher OPG expression levels in breast cancer cells with a basal phenotype, we performed our studies using the MDA-MB-231 human breast cancer cell line. It has no OPG DNA copy gain, but reveals robust OPG mRNA expression in several breast cancer cell line data sets at Oncomine, at R2 (results not shown), and in our own analysis and readily proliferates and metastasizes from the chick CAM.

We used an OPG shRNA plasmid to stably knock down OPG expression in MDA-MB-231 cells. Knockdown clones were selected with puromycin and OPG protein expression levels were determined by enzyme-linked immunosorbent assay (ELISA). After 24-h incubation, a significant reduction in OPG protein expression was observed in OPG shRNA-transfected cells as compared with noncoding shRNA control cells and parental MDA-MB-231 cells (Fig.2B). There was an ∼60% reduction in OPG protein levels in OPG shRNA cells, compared to the cells transfected with the control shRNA.

These transfected cells were used in the chick embryo model to assess the impact of OPG on tumor growth and metastasis. Cells were inoculated on the CAM of 10-day-old chick embryos and incubated for 1 week. Primary tumors were harvested and weighed and a section of the lower CAM (distinct from the inoculation site) and chick liver were analyzed for metastasis by human Alu-specific PCR. OPG shRNA-treated MDA-MB-231 cells formed larger tumors as compared to the shRNA control cells (Fig.2C), but showed less metastasis to the distant CAM and the liver (Fig.2D). Even upon normalization of metastasis data to primary tumor weight, significantly less metastasis occurred per milligram of tumor tissue from OPG shRNA-treated than from control shRNA-treated MDA-MB-231 cells (Fig.2E).

**Figure 3:**
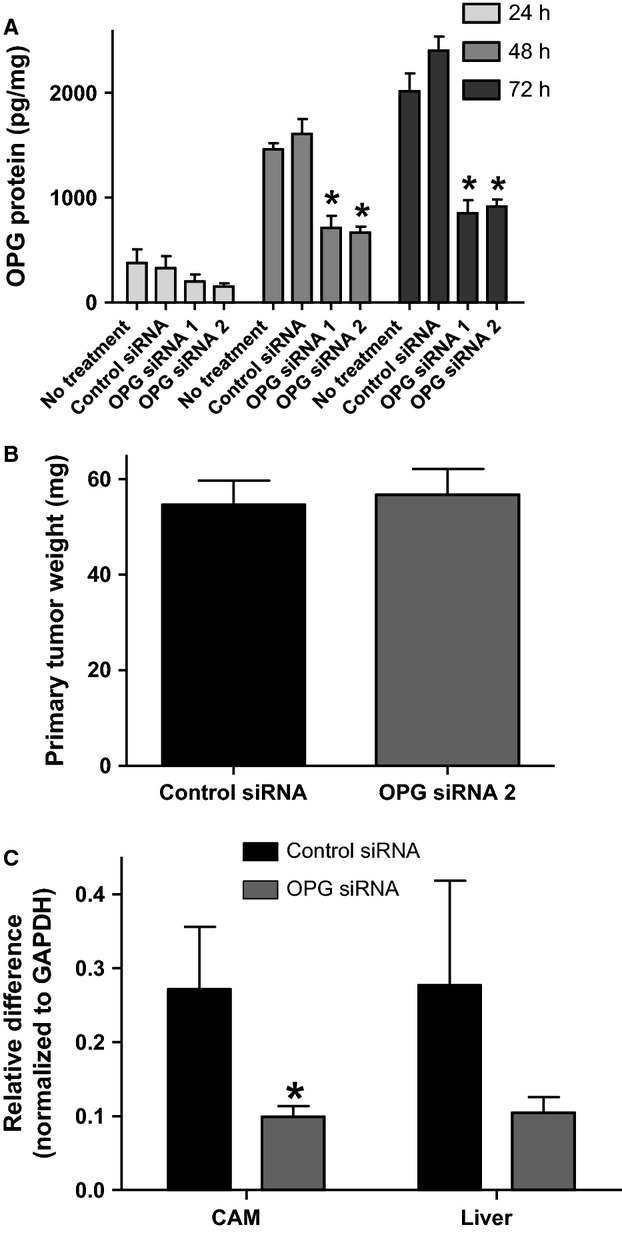
OPG knockdown by siRNA reduces metastasis of MDA-MB-231 cells in the chick embryo spontaneous metastasis model. (A) MDA-MB-231 cells were transfected with two separate OPG siRNAs or with negative control siRNA. OPG expression was measured by ELISA in cell culture supernatants after 24-, 48-, and 72-h incubation (*P < 0.05 significantly different from siRNA control cells, *n* ≥ 3). OPG siRNA #2 or negative control siRNA cells were incubated 1 week in the chick embryo metastasis model and (B) primary tumors were collected and weighed and (C) chick lower CAM and liver analyzed by quantitative PCR for human Alu DNA (*P < 0.05, *n* = 17). All data are represented as mean ± SEM.

We confirmed the specificity of the effect by repeating the experiment using OPG siRNA as an alternative strategy to reduce OPG expression levels in MDA-MB-231 cells. Cells were transfected with OPG or control siRNA, and OPG protein levels measured after 24, 48, and 72 h. Transfection with two separate OPG siRNA constructs led to a significant reduction in OPG protein expression levels compared to control cells for up to 72 h post transfection (Fig.3A). An ∼60% reduction in OPG expression was observed with siRNA transfection, similar to that observed with shRNA-treated cells. We used cells transfected with OPG siRNA construct #2 or control siRNA in the chick embryo metastasis model, as described above. There was no difference in primary tumor weight between OPG or control siRNA cells (Fig. 3B). Similar to OPG shRNA cells, OPG siRNA-treated cells produced significantly lower levels of metastasis in the chick embryo model as compared to control siRNA-treated cells (Fig.3C). Therefore, the data from both types of OPG knockdown experiments strongly support a role for OPG as a metastasis promoter for MDA-MB-231-derived primary tumors.

### OPG expression knockdown in MDA-MB-231 and MDA-MB-436 breast cancer cells reduces metastasis in the chick embryo experimental metastasis model

**Figure 4:**
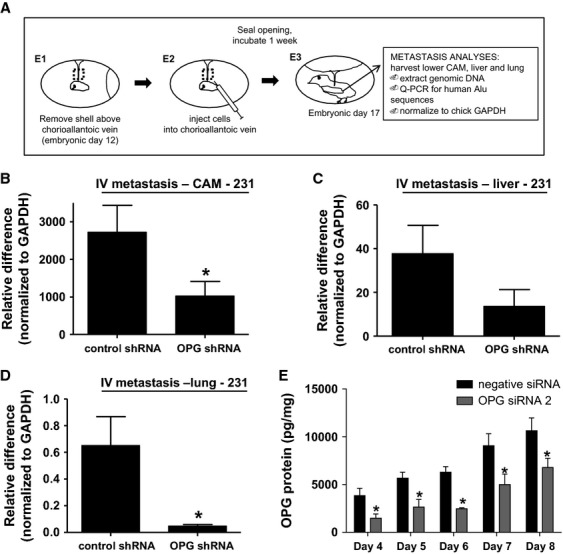
OPG knockdown by shRNA reduces metastasis of MDA-MB-231 cells in the chick embryo experimental metastasis model. (A) Overview of this model. OPG or control shRNA cells were incubated 1 week in the chick embryo experimental metastasis model and genomic DNA from (B) chick lower CAM, (C) liver, and (D) lung analyzed by quantitative PCR for human Alu DNA (*P < 0.05, *n* = 20). (E) MDA-MB-231 cells were transfected with OPG siRNA 2 or with negative control siRNA. OPG expression was measured by ELISA in cell culture supernatants days 4, 5, 6, 7, and 8 after transfection (*P < 0.05 significantly different from siRNA control cells, *n* = 3). All data are represented as mean ± SEM.

To determine whether knockdown of OPG could directly impact metastasis, distinct from effects related to a primary tumor, we used the chick embryo experimental metastasis model, outlined in Figure4A. In this model, a section of the shell is removed at embryonic day 12 (E1) and tumor cells are introduced by intravenous injection into the chorioallantoic vein (E2). The egg is then incubated for 1 week after which metastasis can be quantified by performing quantitative PCR for human Alu repeat sequences in DNA extracted from chick tissue 18. Control shRNA- and OPG shRNA-treated MDA-MB-231 cells were injected into the chorioallantoic veins of 12-day-old chick embryos and incubated for 1 week. A section of the lower CAM (distinct from the inoculation site), chick liver, and lung were analyzed for metastasis by human Alu-specific PCR. OPG shRNA-treated MDA-MB-231 cells had lower levels of metastasis to the distant CAM, lung, and liver (Fig.4B–D). We extended our analyses of the duration of OPG siRNA knockdown in MDA-MB-231 cells to confirm that reduction in OPG protein levels were maintained for the duration of this experiment (Fig.4E). These data are in agreement with the impact on metastasis observed with the spontaneous metastasis model and suggest that OPG can directly impact metastasis distinct from effects at the primary tumor.

**Figure 5:**
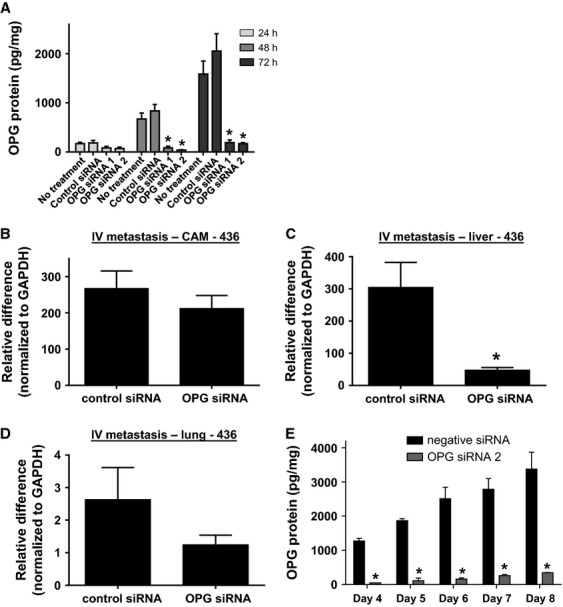
OPG knockdown by siRNA reduces metastasis of MDA-MB-436 cells in the chick embryo experimental metastasis model. (A) MDA-MB-436 cells were transfected with two separate OPG siRNAs or with negative control siRNA. OPG expression was measured by ELISA in cell culture supernatants after 24-, 48-, and 72-h incubation (*P < 0.05 significantly different from siRNA control cells, *n* ≥ 3). (B) chick lower CAM, (C) liver and (D) lung analyzed by quantitative PCR for human Alu DNA (*P < 0.05, *n* = 18). (E) MDA-MB-436 cells were transfected with OPG siRNA 2 or with negative control siRNA. OPG expression was measured by ELISA in cell culture supernatants on days 4, 5, 6, 7, and 8 after transfection (*P < 0.05 significantly different from siRNA control cells, *n* = 2). All data are represented as mean ± SEM.

We performed the experimental metastasis model experiments with a second triple-negative cell line—MDA-MB-436. In vitro analyses confirmed successful knockdown of OPG expression with siRNA in this cell line (Fig.5A). We used cells transfected with OPG siRNA construct #2 or control siRNA in the chick embryo experimental metastasis model, as described above. Similar to the MDA-MB-231 cells, OPG siRNA-treated MDA-MB-436 cells produced lower levels of metastasis as compared to control siRNA-treated cells (Fig.5B–D). We extended our analyses of the duration of OPG siRNA knockdown in MDA-MB-436 cells to confirm that reduction in OPG protein levels was maintained for the duration of this experiment (Fig.5E).

### OPG knockdown does not affect sensitivity to TRAIL-induced cell death

**Figure 6:**
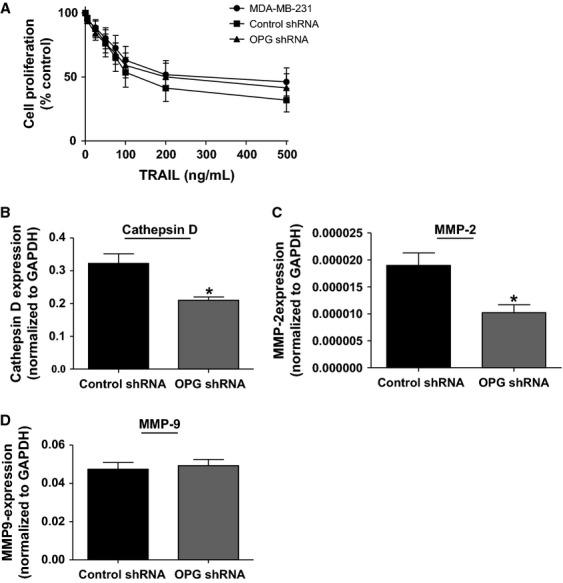
OPG knockdown does not influence sensitivity to TRAIL treatment but leads to a reduction in protease expression. (A) MDA-MB-231, OPG or control shRNA-transfected cells were treated for 48 h with increasing concentrations of TRAIL (0–500 ng/mL), after which cell viability measured by MTT assay. Cell viability is expressed as% proliferation of untreated cells for each condition (*n* ≥ 3). OPG or control shRNA-transfected cells were incubated for 24 h, RNA was extracted and expression of (B) Cathepsin D, (C) MMP-2, and (D) MMP-9 mRNA measured by qRT-PCR. (*P < 0.05, *n* = 6). All data are represented as mean ± SEM.

We went on to investigate the mechanism whereby OPG might impact breast tumor metastasis. Previous in vitro studies in several tumor cell lines have suggested that OPG has tumor-promoting effects by acting as a decoy receptor for TRAIL, preventing apoptosis 11,16. We therefore determined the impact of OPG knockdown by shRNA or siRNA on TRAIL-mediated cell death. Untreated MDA-MB-231 cells, negative controls, stably transfected OPG shRNA cells, or cells transfected with OPG siRNA construct #2 for 24 h were treated with increasing concentrations of TRAIL (0–500 ng/mL). Cell viability was measured by MTT assay after 48 h. Neither OPG shRNA- (Fig.6A) nor siRNA-transfected (data not shown) cells showed increased susceptibility to TRAIL-mediated death compared to negative control cells or untreated MDA-MB-231 cells. This suggests that OPG mediates its effect on metastasis from the primary tumor through a TRAIL-independent, alternative mechanism.

### OPG knockdown leads to reduced protease expression and invasion

To investigate these alternative mechanisms, we analyzed mRNA from OPG- and control shRNA-treated cells using a quantitative reverse transcription PCR (qRT-PCR) array focused on genes involved in breast cancer. We observed a significant reduction in mRNA levels for the protease Cathepsin D in OPG shRNA-transfected, as compared to control shRNA-transfected cells (59.2% mRNA expression, *P* = 0.044; *n* = 3). Separate qRT-PCR reactions also showed this significant decrease in Cathepsin D mRNA expression in OPG shRNA knockdown cells (Fig.6B). We used additional separate qRT-PCR assays to examine the expression of two other proteases, MMP-2 and -9, which also showed reduction in mRNA levels in the array. We found a significant reduction in MMP-2 gene expression upon OPG knockdown (Fig.6C), but did not see changes in MMP-9 mRNA levels (Fig.6D).

**Figure 7:**
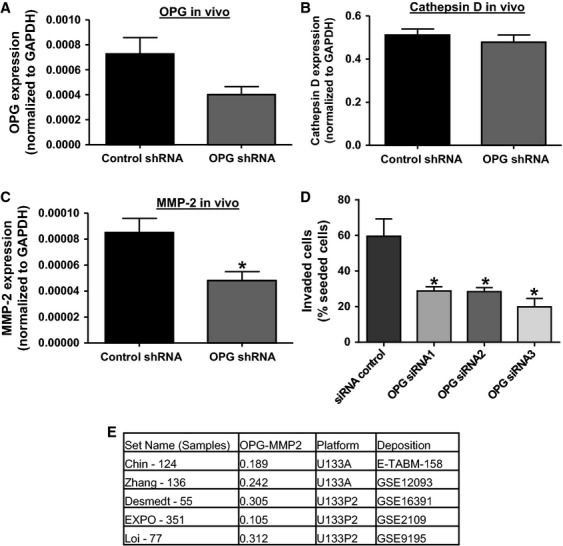
MMP-2 expression is reduced in MDA-MB-231 primary tumors and correlates with reduced OPG levels and invasion capability. OPG or control shRNA-transfected cells were incubated on the chick CAM for 1 week, RNA was extracted from the primary tumors and expression of (A) OPG, (B) Cathepsin D, and (C) MMP-2 mRNA measured by qRT-PCR. Data are represented as mean ± SEM. (*P < 0.05, *n* = 7). (D) MDA-MB-231 were transfected with 3 OPG siRNAs or negative control siRNA for 24 h before seeding on a collagen matrix, and invasion assayed 24 h later. Data are represented as mean ± SEM (*P < 0.05, *n* = 3). (E) Correlation of OPG and MMP-2 mRNA expression in breast cancer data sets in the public domain. Set Name (Samples) indicates the data set used and number of tumor samples in the dataset. OPG-MMP2 indicates the values of a Pearson (2-log) correlation test, all with *P* < 0.05. Platform indicates whether Affymetrix U133A or U133 Plus 2.0 was used. Deposition indicates the source of the data set. For expression value calculations and statistics see Materials and Methods.

We extracted RNA from OPG shRNA and control shRNA-transfected cell primary tumors to determine whether OPG knockdown and its corresponding changes in protease expression were maintained after 1 week of incubation on the chick CAM (Fig.7A). We found that reduced OPG expression in OPG shRNA-derived tumors was still close to significance (*P* = 0.054). Analysis of Cathepsin D and MMP-2 gene expression levels showed that although there was no longer a significant reduction in Cathepsin D expression, MMP-2 expression reduction in OPG shRNA tumors was maintained (Fig.7B and C). To link the alteration in protease expression levels with an impact on metastasis, we performed cell invasion assays with the MDA-MB-231 cells transfected with OPG siRNA. We found that OPG knockdown cells had a reduced ability to invade through a matrix of collagen, a substrate for MMP-2 (Fig.7D). This result is supported by analysis of breast cancer data sets in the public domain, where we found significant positive correlations between OPG and MMP-2 mRNA expression levels (Fig.7E). These data indicate that a reduction in protease activity may mediate the reduction in invasion and thus metastasis of OPG knockdown cells, especially through decreased MMP-2 expression.

## Discussion

We investigated the role of OPG produced by human breast cancer cells in metastasis. OPG gene copy number gain correlates with a poorer prognosis in breast cancer. It also appears that breast cancer cells with a basal phenotype express higher levels of OPG mRNA. We found that knocking down OPG expression in triple-negative breast cancer cells led to a significant reduction in metastasis in the chick embryo metastasis model. A reduction in metastasis was observed from both a primary tumor and by intravenous injection of tumor cells, suggesting a direct impact of OPG on metastasis. The knockdown of OPG expression did not alter sensitivity to TRAIL-induced cell death, indicating that the impact on metastasis occurred via another, novel, mechanism. Gene expression analysis revealed a reduction in expression of the proteases Cathepsin D and MMP-2 in the OPG knockdown cells with reduced levels of MMP-2 maintained after primary tumor growth. We observed that OPG knockdown cells had a reduced level of invasion through collagen, an MMP-2 substrate. Analysis of publicly available microarray data for human breast tumors showed a positive correlation between OPG and MMP-2 expression. Our data suggest that OPG represents a novel regulator of metastasis in human breast tumor cells and requires further investigation as a potential biomarker and therapeutic target.

This is the first study to indicate a metastasis-promoting effect of endogenous OPG production by primary breast tumor cells. In a previous study, MCF-7 breast cancer cells overexpressing an OPG transgene were orthotopically injected into the mammary gland fat pads of nude mice 23. Increased primary tumor growth was observed in OPG overexpressing cells as compared to parental MCF-7 tumors. While this study shows a tumor-promoting role for OPG expressed by the primary breast tumor, the role of OPG overexpression on metastasis was not investigated.

Contrary to published in vitro data 11,24, we did not see an impact on TRAIL-mediated apoptosis upon OPG knockdown. Since both our RNAi-mediated knockdown methods achieved about 60% knockdown of OPG expression, possibly sufficient OPG remained to interact with and block TRAIL-induced apoptosis. Rachner and colleagues also used siRNA to knockdown OPG expression in MDA-MB-231 breast cancer cells 25. They achieved a slightly greater impact on OPG expression with ∼70% knockdown; however, this had a limited effect on TRAIL-induced apoptosis: the cell viability remained above 80%. In addition, similar knockdown of OPG levels in MCF-7 breast cancer cells did not alter TRAIL sensitivity 25. A more recent study investigated the relevance of the OPG-TRAIL interaction using an in vivo murine model of bone metastasis 26. A cell line derived from MDA-MB-231 cells that forms osteolytic lesions after intratibial injection was manipulated to overexpress OPG. While these cells showed resistance to TRAIL in vitro, no impact was observed on sensitivity to TRAIL treatment in vivo after intratibial injection. These findings and our data suggest that the interaction between OPG and TRAIL in primary and metastatic breast cancer is not yet clear and requires further investigation. It should, however, be noted that the TRAIL/OPG interaction has been shown to be significant in other pathologies, particularly cardiovascular disease 27.

Transcriptome changes correlating with decreased metastasis in this study revealed a decrease in expression of the proteases Cathepsin D and MMP-2 in OPG knockdown cells. Lower levels of both these proteases could mediate a decreased level of metastasis. The knockdown of Cathepsin D expression with siRNA in MDA-MB-231 cells has been shown to reduce the ability of cells to form lung metastases in a murine tail vein injection model 28. In patients, higher levels of Cathepsin D in the primary breast tumor correlate with increased incidence of metastasis and poorer disease prognosis 29. Although OPG has not yet been correlated with changes in protease expression in the context of primary breast tumors, correlations have already been shown in other tissues. Treatment of murine aortic vascular smooth muscle cells with OPG led to an increase in gene expression of MMP-2 and MMP-9 30. In the current study, we saw a reduction in MMP-2 expression levels with OPG knockdown, but no significant effect on MMP-9 gene expression. Further investigation is required to determine the mechanism whereby OPG regulates protease gene expression, particularly MMP-2 expression, whose expression levels showed a prolonged decrease. Interestingly, OPG and MMP-2 expression were significantly positively correlated in several breast cancer mRNA expression data sets in the public domain, further supporting a functional role for this signaling axis in breast cancer.

Our analyses of publicly available breast cancer data sets showed a correlation between OPG gene copy number gain and poor prognosis, and a correlation of high OPG mRNA expression and more aggressive breast cancer subtypes. A recent analysis associated a single-nucleotide polymorphism in the OPG gene with increased risk of breast cancer development 31. Both studies suggest that further investigation into the significance of OPG as a biomarker of disease risk and prognosis is warranted. In addition, although this study focused on breast cancer, OPG has been linked with poorer prognosis in other tumor types. In a study of 103 gastric adenocarcinoma tissues, high levels of OPG gene expression correlated with increased invasion and metastasis, and predicted poor prognosis 32.

In conclusion, we have demonstrated a metastasis-promoting role for OPG in human breast cancer cells. This effect is associated with a reduction in invasion and an alteration in levels of the proteases Cathepsin D and MMP-2. This study demonstrates the need for further investigation into OPG as a metastasis regulator and potential biomarker and therapeutic target in breast cancer.
